# Intraoperative Teaching in Robotic-Assisted Surgery—A Systematic Review

**DOI:** 10.3390/jcm15197529

**Published:** 2026-09-28

**Authors:** Anna K. Kieslich, Ruari Jardine, Hussain Ibrahim, Areeg Calvert, Katrina Knight, Kenneth G. Walker, Kim A. Walker, Angus J. M. Watson

**Affiliations:** 1 NHS Highland, Raigmore Hospital, Old Perth Road, Inverness IV2 3UJ, UK; ruari.jardine@abdn.ac.uk (R.J.); hussain.ibrahim@abdn.ac.uk (H.I.); areeg.calvert@nhs.scot (A.C.); katrina.knight3@nhs.scot (K.K.); kenneth.walker@nhs.scot (K.G.W.); 2 University of Aberdeen, Kings College, Aberdeen AB24 3FX, UK; kim.walker@abdn.ac.uk (K.A.W.); a.watson@abdn.ac.uk (A.J.M.W.); 3 Surgical Education and Research Innovation Lab (SERI Lab), Royal College of Surgeons of Edinburgh, Nicolson Street, Edinburgh EH8 9DW, UK

**Keywords:** robotic-assisted surgery, surgical education, training-the trainer, dual console, operative skills, verbal instructions

## Abstract

**Background/Objectives**: Robotic-assisted surgery (RAS) teaching, using simulation, has been examined widely. Less is known about teaching in the intraoperative environment. We conducted a systematic review of skills, strategies and facilities necessary for intraoperative RAS teaching. **Methods**: A systematic review of evidence on RAS curricula and teaching was conducted using MEDLINE, PubMed, Embase, CINAHL and PsycINFO in February 2024, updated in June 2025. A total of 10,000 references were screened for eligibility using the PICO (population, intervention, comparator, outcomes) framework, with a focus on surgeons and intraoperative RAS teaching. Data were extracted and analysed thematically using NVivo 14. Methodological quality was assessed using the MMERSQI (Modified Medical Education Research Study Quality Instrument) criteria and the Joanna Briggs (JBI) critical appraisal tool. The review was registered with PROSPERO under CRD42024566778. **Results**: A total of 16 publications met the inclusion criteria. Most studies were small, used qualitative methodologies and were conducted in general or colorectal surgery. Verbal instruction was the most common teaching method. Force sensitivity, robotic control, including retraction, perception of the field and team management were skills taught intraoperatively. RAS training-the-trainer publications focused on enhancing autonomy. **Conclusions**: The volume of available evidence is low. Trainee autonomy is the main challenge in intraoperative RAS training and may be enhanced by training-the-trainer skills training. Verbal teaching accompanied by a demonstrative gesture appears to be useful. Intraoperative RAS skills taught include third arm retraction, awareness of the field and management of the bedside team. The use of a dual console to facilitate teaching is endorsed by most RAS trainers but is not an absolute requirement for training.

## 1. Introduction

Until recently, robotic-assisted surgery (RAS) training in Europe was focused on established surgeons undertaking industry-sponsored simulation courses and brief proctorships [1].

However, with the expanding use of RAS across clinical practice, surgical trainees are being increasingly exposed to the modality during their training. Various pre-clinical training curricula have been developed for trainees [2,3,4,5,6], and there are now multiple publications on simulation training for RAS [7,8,9].

A major challenge for trainee participation [10] is the transition from assisting at the bedside to operating the console [11], and little is known about how to optimally deliver intraoperative RAS teaching. All the functions of the surgical robot, including retraction, can be controlled from a single console. Console operating has been described as “all-or- none you are either in the driver’s seat, or you are a passenger” [12]. Therefore, there is a risk of trainees taking on spectator-only roles in RAS [13], which may come at the cost of reduced trainee autonomy [14,15,16].

Additionally, the immersive RAS console and the bedside cart physically change the operating room set-up, increasing the complexity of both the equipment and team communications [17]. This creates new demands on the non-technical skills required for the surgical team [18]. RAS teaching may be improved by the availability of a dual console system, which facilitates two-surgeon collaboration by allowing shared control of the operative arms [12] and provides additional teaching tools [19]. The dual console provides virtual pointers, on-screen drawing and various swap modes for the surgical trainer to use [19].

Training skills and the need for training standards are being increasingly recognised as relevant to patient safety in the surgical community [20]. The goals of supervision in the operating room are to maximise trainee participation, transfer expert knowledge (and skills), create a supportive learning environment, facilitate trainee confidence, safeguard patient outcomes whilst also tracking time and the progress of the procedure [21].In addition, the surgical educator must foster psychological safety, provide teaching targeted to the learner and be able to communicate their thought processes to the trainee. These skills need to stand alongside exemplary medical knowledge and good patient care [22]. However, surgical supervisors vary widely in the degree of guidance, critical feedback and autonomy they provide to trainees, which influences how trainees learn to operate [23].

An excellent example of how trainer skills can contribute to the improvement of surgical quality is the Laparoscopic Colorectal Training the Trainer Course (LapCo) [24]. A large part of the success of the LapCo programme can be attributed to its emphasis on training the educators [24,25]. All LapCo trainers received training and an assessment of their teaching skills before being approved to teach laparoscopic surgery to other colorectal surgeons [25]. Trainer-training included the use of task deconstruction, the enhancement of intraoperative instructions and encouragement not to take over the surgical procedure [26].

Many of the training-the-trainer skills from the LapCo programme are of relevance to RAS and are already being employed [27,28,29]. However, the distinctive intraoperative skills necessary for RAS operating and RAS-specific teaching methods are not addressed in the preparation of current RAS trainers [30].

Therefore, unique teaching methods may be necessary to train the next generation of RAS surgeons [14].

The aim of this systematic review, therefore, was to explore the literature on successful intraoperative RAS teaching methods. We focussed this review on intraoperative RAS skills, teaching methods and facilities. We postulated that there are unique aspects to intraoperative RAS teaching, and these could also inform intraoperative teaching in other surgical modalities.

## 2. Materials and Methods

This systematic review contributes to a larger body of work, which has been separated for analytical purposes. The detailed methods have been previously published [31,32].

### 2.1. Search and Sources

This systematic review follows the PRISMA (Preferred Reporting Items for Systematic Reviews and Meta-Analyses 2020) Guidelines [33]. See Figure 1 for PRISMA flow chart and PRISMA checklist in Supplementary Materials. We used Covidence systematic review software (Veritas Health Innovation, Melbourne, Australia, available at www.covidence.org) for title and abstract screening, full-text review and data extraction.

Systematic platform-adapted Boolean literature searches were built with the assistance of an information consultant and a librarian. Searches combined RAS with training and were conducted across six different databases (MEDLINE, PubMed, Embase, Scopus, CINAHL, PsycINFO). Even though PubMed contains MEDLINE, a separate MEDLINE search resulted in 512 additional articles, which we included in the screening. The search was limited to the period from 1997, when the first RAS operations were performed, to current day (2025). Publications in English and German were included. See Supplementary Materials for platform-specific searches.

The searches were conducted between 17 January 2024 and 6 February 2024 after several scoping searches and updated on 20 June 2025. Reference chaining was used to ensure data saturation, and one additional reference was identified.

The search yielded 18,823 references, with 10,000 studies after exclusion of duplicates. Title and abstract screening excluded 8978 studies as irrelevant. 1022 full-text studies were assessed and 172 studies met the general inclusion criteria of the systematic review. (PRISMA Flow-Chart Figure 1) The review was registered on PROSPERO under ID Number CRD42024566778 on 2 October 2024.

### 2.2. Study Selection

We defined study eligibility criteria for this systematic review and analysis using the PICO (population, intervention, comparator, outcomes) framework (Table 1).

We focused on the setting of RAS in the specialties of general surgery, gynaecology, urology and cardiothoracic surgery. These specialties have a comparable operative field in a confined cavity (compared with orthopaedic surgery and ENT (Ear Nose and Throat) Surgery) and use the Da Vinci platform (Intuitive, Sunnyvale CA, USA), as well as other emerging surgical robots such as the Versius (CMR Cambridge, Cambridge, UK) and the Hugo (Medtronic, Minneapolis, MN, USA).

We also excluded articles on learning curves as they were often presented as a case series and primarily did not describe the method of learning or teaching even though they were initially included. AK plus one of RJ, HI, AC or AW did the title and abstract screening. AK plus one of RJ or AW did the full text review. Conflicts were resolved by discussion. Data extraction and analysis were done by one reviewer (AK) and cross-checked by another (AW) to reduce bias. Quality assessment of qualitative studies was done by one reviewer (AK) and sense-checked by another to reduce bias (KAW). We identified 26 publications which considered intraoperative teaching. To aggregate the evidence on intraoperative teaching, we narrowed our inclusion criteria for the analysis and excluded 10 further papers. Of the excluded papers, three papers [34,35,36] focused on developing modular steps for radical robotic prostatectomy (RARP), two papers focused on educational video resources to increase intraoperative console participation [37,38], one study considered theatre scheduling to maximise exposure [39], two studies explored the functionality of the dual console, without providing details on teaching methods [12,40], one study examined the functionality of a ghost tool, which is currently not widely available [41], and one qualitative study addressed a broader question of implementing teaching for trainees [42]. Structured modular teaching has been identified as an important method for intraoperative RAS teaching [31,32]. The studies considering modular training developed and validated RARP-specific modules without discussing which RAS skills were necessary for module achievement or details of teaching methods necessary for module application [34,35,36]. Therefore, these studies were deemed to be not comparable to other studies and were excluded from further analysis.

### 2.3. Data Extraction and Analysis

The papers included in the systematic review were heterogeneous, as a large volume of publications met our inclusion criteria. We therefore split the systematic review into distinct topics and analysed studies with comparable methodology and outcomes together. In this manuscript, we describe the analysis of intraoperative RAS teaching studies.

The following questions were used to conduct a thematic analysis of the included papers:

1. What are the specific barriers in RAS training?
2. What are the unique skills that need to be taught intraoperatively in RAS?
3. What are the unique teaching methods necessary for successful RAS teaching?
4. Are specific facilities needed for RAS teaching?

Coding was undertaken line by line in an iterative process. The codes were aggregated (collected) under the relevant questions. Overlapping codes were grouped together and included under the relevant question during analysis. Coding and analysis were conducted with Lumivero (2023) NVivo (Version 14), (available at www.lumivero.com).

Quality assessment of the studies was undertaken according to study methodology. We employed the Joanna Briggs Institute critical appraisal tool (JBI) and the Modified Medical Education Research Study Quality Instrument (MMERSQI) tool. We considered the rounds conducted, number of participants, response rate and Cronbach’s alpha for the Delphi studies on expert opinion. (see Table 2).

One study employed the commonly used and validated Global Evaluative Assessment of Robotic Skills (GEARS) domains as a framework to observe intraoperative instructions and assess trainee skills [43]. GEARS had originally been adapted from Global Assessment of Laparoscopic Skills (GOALS) score, which had been validated and shown to reliably distinguish laparoscopic skills [44,45]. GOALS incorporates the domains depth perception, bimanual dexterity, efficiency, tissue handling and autonomy. GEARS additionally includes ‘robotic control’ pertaining to instruments and camera. The GOALS domain ‘tissue handling’ has been renamed ‘force control’ due to the lack of tactile feedback with RAS [46]. We considered the skills described in the other examined studies under the GEARS framework where appropriate. Identified skills that did not align with the GEARS framework were explained separately.

**Table 2:** Summary of included studies.

Study	Topic/Aim	Methodology	Findings	Examples	Credibility/Quality
Anand [43] et al. 2024 USA Multispecialty (61% Minimally invasive surgery/small cases General surgery)	Facilities and audio coaching, Single vs. dual console intraoperative coaching and resident autonomy.	Survey and Qualitative examination of audio (conductive coding of audio); Wisconsin Surgical Coaching Rubric WiSCoR, GEARS, NASA-TLX of faculty	18 cases (7 single console, 11 dual console); 11 trainer–trainee pairs; survey 18 trainees, 11	No difference found in resident autonomy score and faculty coaching score single vs. dual console Majority of statements force sensitivity and robotic control No evaluation of faculty NASA TXL provided by console type Gap in coaching self-reflection	Low
Beane [10] 2019 USA Urology	How trainees succeed when new technologies limit legitimate peripheral participation, RAS vs. open surgical teaching	Multisite ethnography in Urology open vs. RAS and semi-structured interviews focusing on trainees who succeed in RAS;	2 years, 94 procedures, 478 h observation, 62 interviews 18 surgeons, 22 team 16 residents	Barriers: no peripheral participation, Visible mistakes, Helicopter training, trainer independence from trainees Shadow learning of successful trainees: ‘‘premature specialisation’’; ‘‘abstract rehearsal’’ (Simulation and video) ‘‘under-supervised struggle,’’	Moderate ethnography without researcher positionality
Brian et al. [47] 2024 USA General surgery	Trainer skills: how instruction manifests in the robotic operating room and how observed robotic instructional practices map with trainees preferred robotic instructional practices	Constructivist framework, co-creation of the learning experience during case observations and the meaning that interviewees made about their robotic surgical experiences.	38 faculty and fellows 10 cases, 20 interviews	Suboptimal instructional practices: Contextualisation without explaining reason Individualisation without consideration of trainee level Trainer cannot explain what is to be done and takes over Multimodality-clarity is provided with addition of gesture	High
Cristofari et al. [48] 2022 Switzerland Bariatric surgery	Shed light on surgeons’ training at the dual console as it happens during actual surgeries, and to identify didactic difficulties that may be resolved with targeted effort.	Observation and Interviews, Filming (3 cases) and audio recording, Self-confrontational Interviews with edited excerpts,	2 surgeons; interviews with one lead surgeon, one trainee participated in all 3 cases	Skills described: Managing the (bedside) team Using the third arm Perception of the operative field Instructions: Physical separation requires different instructions More specific instructions necessary	Moderate
Collins et al. [49] 2019 Europe Multispecialty	Define the key elements within a Train-the Trainer programme, Panel with expertise in training from both the healthcare and the military and airline industries.	Literature Review; Discussion at Meeting; 3 Round Delphi 80% for consensus; Participants from USA and eight European countries.	Voting: 28 participants (24 surgeons 4 healthcare industry) Meeting: 24 surgeons, 8 healthcare industry, academics, or military or airline industry personnel with expertise in training.	100% agreement on need of TTT course, clearly defined selection criteria, delegates should be tested, agreement of goals and objectives, learn when to take over, TTT with opportunities to practice in the operating room and laboratory/simulation setting, know how to shorten the learning curve.	Delphi, 80% agreement, 3 rounds, 82% (23/28) response rate first round, 89% (25/28) 2nd and 3rd rounds: Cronbach alpha 0.90
Eardley et al. [50] 2020 UK Colorectal Train-the-Trainer	Develop and evaluate a train-the trainer course	Course based on JAG and LapCo train the trainer, Pre- and post-course questionnaire, evaluate by self-assessment of learning	8 delegates from across Europe, 4 faculty	Three delegates received no previous train-the trainer teaching. Delegates increased their knowledge of course objectives and identified learning points for changed practice. Delegates highlighted “no common language” for teaching.	MMERSQI 45
Gómez Ruiz et al. [51] 2019 Spain; Colorectal	Train-the trainer Establish expert consensus on the requirements for a robotic TTT curriculum amongst robotic surgeons and trainers.	literature review and two brainstorming sessions, 3 Delphi rounds 80% consensus,	Participants: 14 colorectal surgeons, selected based on their expertise in training and in robotic surgery, min 50 cases. (one UGI surgeon)	Conscious competency Taking over in theatre Performance-enhancing instruction Dual-task interference Optimising communications and training environment, Reflection on training, Dealing with difficult trainees Optimising feedback	Delphi, 80% agreement 3 rounds, Response rate: first and second rounds was 100% (14/14) and for the third round 93% (13/14). Cronbach alpha > 0.8
Green et al. [52] 2019 USA Colorectal	Determine what educational surgical themes from microanalysis of intracorporeal robotic footage from a robot-assisted surgical procedure.	Video and audio from 6 h video edited to 35 min, repeated review and qualitative memo, Microanalysis of selection of 2 min clip in 1 s intervals	Analysis of 2 min of 6 h colorectal case, 1 RAS surgeon one trainee by the bedside	Observation is based on an incomplete visual framework. Change in operative focus occurs during in adequate tension, bimodal manipulation and hand-movements in console are not appreciated when watching the screen	Moderate
Green et al. [53] 2020 USA General surgery	Characterise the instructional techniques used in the robotic teaching environment	Observation and video of instructional sessions in porcine simulations, focus group trainees and trainers, qualitative content analysis,	6 surgical residents, 9 instructors (5 faculty and 4 residents peer-to-peer)	Behaviours commonly used: Verbal direction (explanation of, thought process, compliment) New teaching behaviour: disengaging the residents from the console Instructors focus group: Importance of language. Managing autonomy	High
Green et al. [54] 2023 USA General surgery	Identify aspects of robotic technology that could be targeted to address its inefficiency	Qualitative analysis of experts commenting on Video showing two sets of clips with average performance and mistakes	17 surgeons; male (82.4%), 47 yrs old (SD = 6.6), 13.2 yrs of teaching experience (SD = 8.23) years of teaching experience, academic hospitals (64.7%), 643 RAS cases (SD = 467)	Areas for improved efficiency: Case progression, Robotic capabilities, Instrumentation.	High
Green et al. [55] 2022 USA, General surgery	Investigated the language of perceptual expertise used by robotic surgeons.	Qualitative analysis of experts commenting on Video showing two sets of clips with average performance and mistakes	17 surgeons; male (82.4%), 47 yrs old (SD = 6.6), 13.2 yrs of teaching experience (SD = 8.23) years of teaching experience, academic hospitals (64.7%), 643 RAS cases (SD = 467)	Visual comprehension: tissue structures, anatomic landmarks, adequate field of view. Surgical technique: discussing dissection, tissue handling, instrumentation choice and exposure. Risk Avoidance: Overarching theme for visual comprehension and surgical technique	High
Harji et al. [30] 2025 UK + Ireland Colorectal surgery	Reach an in-depth understanding of the delivery of current proctored training regarding responsibilities and challenges	Online Qualitative focus group of RAS proctors along predefined topics, framework analysis, line-by-line coding	10 proctors, >60% had been robotic trainers for more than 6 years and 60% were considered high-volume proctors (>20 cases annually)	Proctor selection by industry, based on arbitrary criteria. Aim of proctoring is the safe use of the robotic technology. Position as proctor often allows a wider sphere of influence within the surgical community “Sign off” is based on a small number of cases, i.e., 1–5	Moderate
Leon et al. [56] 2022 USA Gynaecology	Investigate surgical education metrics to objectively highlight the advantages of robotic dual versus single console gynaecologic surgery training	Analysis of Fellow participation in hysterectomies with single Vs. dual console; Console times, comparison among individuals and console used	3 fellows, 5 attendings; 126 hysterectomies	Longer fellow console time (*p* < 0.001, Figure 1), more steps (*p* = 0.009), more control switches (*p* < 0.001) for dual console compared to single console cases. No difference in docking time (*p* = 0.15), case time (*p* = 0.79), or complications (*p* = 0.30).	MMERSQI 53.5/85
Satchidanand et al. [57] 2021 USA Urology	Explore the use of microanalysis to describe and evaluate communicative efficiency in RAS through examination of referencing in surgical tasks	Video microanalysis of a teaching situation in RAS; Descriptive taxonomy for referencing strategies in RAS was developed.	Selection of 46 references from 25 exchanges from 100 timepoints of 10 verbal transcriptions from 79 RAS surgeries, number of surgeons involved not specified	Referencing strategies for communication were: Verbal, Gesture, Integrated communication (verbal + gesture). Integrated most common and most successful.	High
Wang et al. [58] 2024 USA General surgery	Understand attending surgeon motivations in granting trainees Individual Console Time (ICT-time actively steering the console) in RAS	Sequential explanatory mixed-methods approach was undertaken to explore the ICT results from RAS console cases with thematic analysis of secondary qualitative interviews with surgeons.	420 RAS cases 30 residents and 9 attending surgeons	Residents may get less active time in RAS than other modalities. ICT significantly increased with seniority (junior 26.8%; senior 42.4% average ICT (*p* < 0.001).) Variability with procedure type and trainer work experience. Surgeons perceived operative time pressure inversely affected resident ICT.	High
Wong et al. [59] 2023 USA Urology	Create a novel classification system to characterise surgical feedback for usefulness	Transcribed and analysed video and audio of RAS intraoperative feedback while trainees on the console Grounded theory, constructivist methodology	29 robotic teaching surgical procedures were recorded, with 3711 instances of feedback 4 attending surgeons, 6 fellows, and 5 residents	Feedback: Anatomic, Procedural, technical, gesture for visual aid technical, praise, criticism Trainee Response: verbal acknowledgement, behavioural change, asks for clarification. Types of feedback associated with variable responses.	High

## 3. Results

Sixteen publications met the inclusion criteria for intraoperative teaching (Table 2).

Studies were conducted in general surgery (n = 5), colorectal (n = 4), urology (n = 3), multispecialty (n = 2), gynaecology (n = 1) and bariatric surgery (n = 1). The work was performed in the USA (n = 11) and Europe (n = 5). The methodology used was qualitative (n = 11), quantitative (n = 1), mixed methods (n = 2) [50,58] and Delphi expert consensus (n = 2). The qualitative methods used were multicentre ethnography (n = 1) [10], qualitative analysis of instructional audio files (n = 1) [43], video analysis (n = 4) [48,52,57,59], confrontational interview (n= 3 [48,54,55], survey (n = 1) [50], focus groups (n = 1) [30], and interviews (n = 2) [47,58]. The topical focus of the studies was dual console (n = 2) [43,56], training-the trainer courses (n = 3) [49,50,51], RAS intraoperative instructions (n = 5) [47,48,53,57,59], RAS proctoring (n = 1) [30], intraoperative console time of trainees (n = 1) [56], RAS training vs. open surgical training n = 1 [10], observational learning (n = 1) [52] and expert language on RAS and efficiency (n = 2) [54,55].

The quality of over half of the qualitative studies was moderate to high (MMERSQI Table 2, JBI Tool, Table 3). Researcher positionality and influence were not addressed in any of the studies, with minimal information reported regarding the researcher in only four studies [47,53,54,55]. Several included qualitative studies originate from the same research team at the University of California San Francisco. This may have created a dominance of teaching culture from one institution and limited the generalisability of findings [47,52,53,54,55]. All studies providing information on researcher positionality originated from this one institution. All Delphi studies included three anonymous rounds and reported consensus at 80%, with an interrater reliability of >0.8 with a response rate of over 80%. The number of participants was low: 14–28 experts. The quality of the quantitative studies was moderate to low (MMERSQI score Table 2). No studies are available examining intraoperative teaching in relation to surgical skill or improved patient outcome measures.

### 3.1. What Are the Specific Barriers for RAS Training?

#### 3.1.1. Trainer Trust

One of the main challenges surrounding RAS teaching involves trusting the trainee to operate the console [10,48,50,53,58]. The reasons suggested for this lack of trust included the serious consequences [10,48] and the exaggerated visibility of mistakes [10], time and cost pressures [58], RAS being a new technology [10,58], the pressure to innovate [10] and instructor specialisation [10]. Additionally, specific RAS features, such as the ease of taking over the dual console [50] and trainer independence from trainees, were thought to be other potential barriers [10,53,58]. RAS surgical trainers were described as engaging in ‘helicopter teaching’, micromanaging every action that the trainees took on the console [10].

The reviewed publications suggested this reduced entrustment was associated with a risk of creating incompetent surgeons in the future [10,53,58]. In addition to the difficulties articulated above, international delegates at a RAS training-the-trainer course had not previously received instructions on how to teach and were not familiar with the training-the-trainer concept generally [50]. No standardised pedagogical training was provided to current RAS proctors [30].

**Table 3:** Quality assessment of qualitative studies (JBI Critical Assessment Tool for Qualitative Research) [60].

Study	Congruity Between Philosophical Perspective and Research Methodology	Congruity Between Methodology and Research Question	Congruity Between Methodology and Data Collection	Congruity Between Methodology and Data Representation and Analysis	Congruity Between Methodology and Interpretation of Results	Information of Cultural or Theoretical Position of Researcher	Influence of the Researcher on the Research Discussed	Adequate Representation of Participants and Their Voices	Ethical Approval	Conclusions Flow from the Data Analysis	Subjective Global Assessment of Quality
Anand [43] 2024	No	No	No	Yes	No	No	No	No	No	Unclear	Low
Beane [10] 2019	Yes	Yes	Yes	Yes	Yes	No	No	Yes	No	Yes	Moderate
Brian [47] 2024	Yes	Yes	Yes	Yes	Yes	Minimal	No	Yes	Yes	Yes	High
Cristofari [48] 2022	Yes	Yes	Yes	Unclear	Unclear	No	No	Yes	No	Unclear	Moderate
Green [52] 2019	Yes	Yes	Yes	Yes	Yes	No	No	No	Yes	Yes	Moderate
Green [53] 2020	Yes	Yes	Yes	Yes	Yes	Unclear	No	Yes	Yes	Yes	High
Green [55] 2022	Yes	Yes	Yes	Yes	Yes	Unclear	No	Yes	Yes	Yes	High
Green [54] 2023	Yes	Yes	Yes	Yes	Yes	Unclear	No	Yes	Yes	Yes	High
Harji [30] 2025	Yes	Yes	Unclear	Yes	Yes	No	No	Unclear	Yes	Yes	Moderate
Satchidanand 2021 [57]	Yes	Yes	Yes	Yes	Yes	No	No	Yes	Yes	Yes	High
Wang [58] 2024	Yes	Yes	Yes	Yes	Yes	No	No	Yes	Yes	Yes	High
Wong [59] 2023	Yes	Yes	Yes	Yes	Yes	No	No	Yes	No	Yes	High

#### 3.1.2. Limited Opportunities for Participation

When trainees are not operating the RAS console, they lose the ability to actively participate in the operation [10]. The physical separation of trainer and trainee, as well as the split of the operative fields (console/bedside) has reduced the instances of direct interaction and observation [53]. The narrow view of the operative field produced by the RAS camera provides trainees with an incomplete understanding of what movements are necessary to achieve good tissue exposure. Novice trainees, especially, may not perceive important actions, such as continued traction or optimised exposure using the third RAS arm, from observation alone [52]. Trainees can see the result of retraction, but not how it is achieved. Learning from observation may therefore be impaired in RAS compared with other surgical modalities, if it is not accompanied by specific verbal instructions [52]. In addition, the opportunity for trainee-to-trainee teaching may be reduced due to the presence of the surgical trainer in the operating room [10]. (Figure 2).

### 3.2. What Are the Unique Intraoperative Skills That Need to Be Taught in RAS?

#### 3.2.1. GEARS Domain Skills

One low-quality qualitative study examined the intraoperative instructions given by trainers and structured them using the GEARS framework [43]. Force sensitivity and robotic control were found to be the focus of verbal teaching [43]. These two skills were also deemed important by experts reviewing videos of average RAS performances and mistakes [43,54,55]. There were fewer verbal instructions about bimanual dexterity [43], but bimodal manipulation to obtain optimal exposure was emphasised by some [52].

Similarly, only a few instructions regarding operative efficiency were observed and the examples provided in this study were not illustrative of this skill [43]. RAS experts identified case progression, robotic capabilities, and instrumentation usage as relevant for efficiency and reacted with annoyance when they observed repetitive movements [54]. These extraneous movements caused extended operative times and were interpreted as being a result of cognitive overload [54]. Several publications mentioned RAS skills that are not specifically outlined in the GEARS domains [43,48,50,52,54,55].

#### 3.2.2. Adequate Tension

Maintaining adequate tissue tension to facilitate dissection is key in any surgical modality. Trainee RAS surgeons need to learn to maintain tissue tension by using the robotic third arm effectively [48,54]. This requires positioning the third arm so it does not obstruct the operative field [48]. Retraction of tissues is more difficult in RAS compared to other modalities because there is a lack of tactile feedback [55] and there is a restricted visual field [52]. If tissue retraction is not adequate, this requires an alteration of the operative field of view, to visualise the instruments so that adequate tissue tension can be re-established [52]. Experienced RAS surgeons frequently picked up on ineffective tissue tension when evaluating videos with suboptimal performance [55].

#### 3.2.3. Perception of the Operative Field

The importance of having a visual perception of the operative field featured in multiple qualitative studies [43,48,52,54,55]. Visual perception includes an understanding of the visualised anatomy [55], the instruments in the operative space [48,55], and an awareness of what is happening in the areas not visualised by the RAS camera [52,54].

#### 3.2.4. Team Communication and Dynamics

The management of the bedside assistant using adequate verbal communication and physical gestures was identified as a skill which must be learned by training on the second console. This training was found to be necessary due to the surgeon’s inability to “read the atmosphere of the team” when operating in the immersive console. Team management from the console can be especially challenging if the bedside assistant is a novice. The lead console surgeon must be able to make decisions with the limited visual and verbal information available [48]. Therefore, it was considered necessary for RAS trainers to be trained and assessed in RAS-specific non-technical skills [50] (Figure 3).

### 3.3. What Are the Unique Teaching Methods of RAS Teaching?

#### 3.3.1. Granting Autonomy

Multiple publications discussed trainer trust and giving trainees autonomy on the RAS console [10,43,47,48,49,51,53,58] with some trainers appearing to grant autonomy by not supervising their trainees at all [10,47].

Others suggested:

- reinforcing feedback when residents are on track,
- providing coaching through a difficult part of the operation before taking over,
- providing reasons for the takeover,
- taking over “briefly in a setting of insurmountable struggle” and handing back control,
- keeping residents engaged during periods of observation [47].
- practice in a RAS training-the trainer course may reduce unnecessary takeover [49,50,51].

Even in the challenging environment of unstructured RAS training, individual trainees were able to develop exceptional RAS skills by engaging in self-driven practice of “premature specialisation”, “abstract rehearsal” and “unsupervised struggle” [10]. Successful trainees were found to increase their robotic skills by engaging in extended simulation training and watching surgical videos, which subsequently increased their console time. Trainees increased their exposure to RAS by prioritising it over other experiences.

“I specifically said [to a new resident] you have to use the simulator. You must use it. When you get on, you want to be there because otherwise they’re [the AP] going to pass you up. So, if you’ve had hours in the simulator, you’re going to look like you know what you’re doing, and they’ll let you do more. Well, I let her get on one, one of these cases, and she looked like she knew what she was doing, and the attending let her go, and she did well (Resident)” [10] (Figure 4).

#### 3.3.2. Verbal Teaching

Verbal teaching was the main method of instruction used [10,48,49,50,51,53]. Finding the right words [50,53,55] and appropriate intensity for verbal instructions was identified as being a challenge for RAS trainers [10]. “Integrated communication” using verbal instructions together with a gesture (e.g., with pointer hands, instruments or screen telestration) was found to be the most frequently used, the most effective [57,59] and liked by trainees [47].

Practice of verbal performance-enhancing instructions was included in training-the-trainer courses [49,51]. Trainees welcomed contextual explanations of instructions and an explanation of thought processes during periods of observation [47]. The adjustment of instructions to take into account trainees’ experience and cognitive load was mentioned in several qualitative publications [10,47,48,50,51].

Debriefing was identified as an option [48,49] to supplement verbal teaching without increasing the intraoperative cognitive load [48]. This also formed part of the training-the-trainer course curricula [49,50,51] and was suggested to be supplemented with video review [49,51]. One low-quality study found there were few prompts to encourage trainees’ self-reflection and suggested these should be increased [43].

To improve the trainer’s verbal instructions, training-the-trainer courses included teaching on conscious competency [50,51], dual-task interference [51], optimising the training environment [49,51], reflection on training [50,51] and dealing with difficult trainees [49,50,51]. Trainers and delegates suggested that developing a RAS-specific teaching taxonomy may be useful [50]. (Figure 4).

#### 3.3.3. Disengaging from the Console

When RAS teaching was examined using a framework on teaching behaviour [61], a unique action was discovered. Trainers asked trainees to disengage from the console for a variety of reasons. Some instructors wanted to draw the trainee’s attention to something happening at the bedside, off the camera screen or to illustrate a specific movement [53].

“We can’t do anything for them. They have to do everything. And I have found a couple of times that I would ask them to take their head out of the console so we could talk about things that occur off screen.” [53]. (Figure 5).

### 3.4. Are There Specific Facilities Necessary for RAS Teaching?

The dual console is deemed important [10,43,53,56,58]. It is accepted as a useful tool for intraoperative instruction [56,58], and is an absolute requirement for some instructors [53]. Trial work comparing single with dual console teaching was affected by some trainers refusing to teach on the single console [43]. In a quantitative study on RAS hysterectomy cases, no significant differences between the two groups (single/dual console) regarding docking time (*p* = 0.15), case time (*p* = 0.79), or complications (*p* = 0.30) were found. However, when the dual console was used a significantly longer console time (*p* < 0.001) and a greater number of steps performed by fellows were found (*p* = 0.009). Additionally, console control was handed over significantly more often between the fellow and attending (*p* < 0.001) in dual console compared to single console operations [56].

Feedback and instructions appeared to differ between dual and single console systems [43]. On the single console, trainees received more constructive feedback, whereas on the dual console sharing of responsibility was seen more often [43]. However, the quality of the qualitative study examining this phenomenon was low. Publications regarding the training-the-trainer courses maintained that dual console teaching should be maximised [48,59]. Other facilities which were mentioned were a safe stress-free training environment [53,62] with good sound-transmission between consoles [48], user machine interface training [51], telementoring [51] and access to simulator and operative video [10].

## 4. Discussion

The aim of this systematic review was to examine the skills, methods and facilities necessary for intraoperative RAS teaching. The use of verbal teaching, the facilitation of trainee autonomy and the use of dual console equipment for skill acquisition were found to be the primary requirements.

Whilst simulation is an established teaching modality in RAS, intraoperative teaching remains necessary to help surgeons attain independent practice [31,63]. Simulation curricula are effective at targeting specific tasks such as wrist articulation, clutching and suturing [64,65,66] but it is the live operative environment where trainees master procedural skills [15,31,67]. However, which RAS skills need to be the focus of intraoperative training remains unknown.

This review has identified three critical intraoperative RAS skills: retraction with the third arm, perception of the operative field and bedside team management. The challenge of adequate retraction, which is augmented by the lack of tactile feedback, is increased in multi-quadrant operating. In this review, all the publications that mentioned retraction are from the field of general surgery [48,52,54,55]. Furthermore, mastering field perception requires the surgeon to maintain awareness beyond the limited area of camera focus. This means accounting for the second and third arms if they are not in view and any additional instruments introduced by the bedside assistant, as well as being aware of what is happening to the patient at the bedside. Novice surgeons have difficulty commanding all three and may initially choose a less focused camera view to maintain control [54,68]. The taxonomy of the GEARS score does not fully account for all the identified skills [45,46,69].

The necessity for the surgeon to control what goes on by the bedside can be reduced but not completely alleviated by an experienced team [70]. The skill of managing the team from the immersive console and providing adequate, goal-directed instructions requires additional non-technical surgical skills beyond those needed for traditional surgical modalities, and can directly affect procedure efficiency [71]. Although non-technical skills teaching is receiving increasing attention in the surgical educational community [31], it has not been formally integrated into most of the published RAS curricula [32].

Verbal teaching is the main method employed by RAS trainers to convey their expertise to trainees [10,48,49,53]. All publications related to RAS training-the-trainer courses included specific attempts to improve verbal “performance enhancing instructions” and feedback [49,50,51], similar to instructions for laparoscopic surgery in the LapCo train-the-trainer programme [25,26,27]. In this review verbal instructions were found to be most effective when accompanied by a gesture [47,57,59], whether with instruments during a brief takeover or with on-screen pointers [57,59]. The functionalities of RAS platforms improve the ease of using gestures both in single and dual console teaching [19] and other advanced illustrative features like ghost tools [41].

The challenge of achieving trainee autonomy during intraoperative training is fundamental to any teaching developments [11]. Excellent surgical educators have been found to teach out of a sense of duty, for intrinsic enjoyment of teaching, for the satisfaction of seeing the development of young colleagues and as a driver for staying up to date themselves [72]. The robot’s functionality provides lead surgeons with independence from assistance and reduces active trainee participation when they are not the console surgeon [11]. This means that surgical trainers do not need the participation of trainees for successful operating. Teaching and learning do not happen ‘by chance’ but only occur if they are provided deliberately at a ‘cost’ to the trainer. It has been acknowledged that the trainee affects the quality of intraoperative teaching through behaviour and preparation [73,74]. However, the effect of trainer and trainee interdependence [75] on the quality of teaching has not been examined in the surgical educational literature. The independence provided by RAS to senior surgeons could alter the unwritten contract in the surgical apprenticeship relationship and reduce the willingness to teach [72].

Trainer trust is not a fixed entity. Training-the-trainer skills practice has been proven effective in laparoscopic colorectal training [24]. The training includes a focus on safely avoiding takeover and the provision of useful actionable instructions at an individual level [25]. These techniques could be applied to RAS-trainer-teaching [49,50,51].

RAS experts have described structured modular training for the intraoperative teaching component [31]. Modular training could be an important method to support trainer trust and help grant autonomy [31]. During modular training, trainees complete operative steps with rising complexity instead of completing the steps of an operation in chronological order [36]. This may offer graduated intraoperative autonomy to trainees appropriate to their abilities and aid the development of trainer trust. Operative steps for RARP have been identified by their difficulty for achievement to provide a structured training approach [34,35,36] and developed as assessment methods for colorectal surgery [76,77,78]. Publications provide procedure-specific checkpoints and timing as methods to recognise achievement of modules. Standardised modular training has been incorporated into RAS curricula, but it is not in widespread use in all RAS specialties [32].

One of the publications in this review identified a novel RAS-specific teaching method which involved disengaging the trainee from the console in order to deliver a microteaching episode. Trainers asked trainees to “come out of the console” to illustrate a point or highlight an occurrence by the bedside. This was observed during a single console simulation event [60]. Out-of-console teaching could serve as an opportunity for trainers to teach in private (off the console speaker phones), reduce the cognitive load for trainees or even ensure the message given has been understood. This teaching method has not been studied exhaustively.

The dual console is the primary physical facility deemed necessary for intraoperative RAS teaching. RAS educators feel strongly in favour of the dual console [43], though the available evidence comparing dual and single console operating is limited and inconclusive [44,51,57]. The dual console may be an important device to support trainer trust by providing ease of takeover, and on-screen pointers to enhance verbal instructions. The collaborative mode on the dual console enables trainers to provide exposure of the field with the third arm, as trainees gradually learn live operating [12].

However, the ease of takeover on the dual console has also been suggested as a risk to trainee autonomy, by reducing the hurdle for trainers to take over [50]. Teaching did occur on single consoles, and even with the trainer at the bedside [48]. Notably, more actionable feedback instructions were observed when the single console was used [44]. We therefore cannot conclude that the dual console is an absolute requirement for intraoperative teaching, especially with senior RAS trainees at the stage of gaining experience in case-mix and procedural efficiency.

The train-the-trainer publications mention the importance of video review [49,51]. Several studies used video for research purposes, and video review is repeatedly mentioned as beneficial for learning in the RAS educational literature [31] but there is little information on how video review may aid intraoperative teaching and feedback. The lack of video use for teaching in most of the included studies may be due to the focus on intraoperative observations. Current fast-moving developments of deep learning models may allow the incorporation of personalised feedback for self-review or aid trainer-trainee video debriefing in the future [79,80]. Additionally, automated assessment metrics provided by future iterations of RAS platforms may provide guidance for the simulated as well as intraoperative environment [62,81].

A strength of this systematic review is the breadth of the search and the unique systematic review question focusing on RAS intraoperative teaching. To our knowledge, the RAS skills which must specifically be addressed during intraoperative teaching have not previously been addressed in this way. The inclusion of qualitative methodology in our analysis highlights some important aspects, which are not easily studied using quantitative methods alone.

The weakness of our systematic review is the quality of evidence available. The level of evidence is low, relying on qualitative or mixed methods studies, expert opinion and a few medium-quality quantitative studies. The qualitative studies are small, dominated by one research team, and they may be specific to one local culture of teaching. Our quality analysis of studies revealed little information on researcher positionality in all the examined publications. This lack of information reduces the generalisability of the conclusions.

Our own ongoing ethnographic research on intraoperative RAS teaching may have influenced our analysis of the available literature. For example, the practice of “teaching outside of the console” has been observed in our study. We have highlighted it here even though this may not be a method in widespread use.

Structured modular teaching is an important teaching method discussed in the RAS educational literature [31,32]. Due to the limited number of publications on modular steps, the procedure-specific nature of reported outcomes, and the sparse detail on how modular steps were applied in teaching [35,36,76], this literature could not be meaningfully synthesised in our systematic analysis.

RAS-specific train-the-trainer courses are currently being developed [48,58,59,62]. However, more research is necessary to ensure the courses optimally address RAS-specific teaching challenges and to optimise teaching with this modality. The publications regarding these courses did not specify which skills need to be taught, and little information was provided on how to overcome RAS-specific teaching challenges. The courses described do address verbal teaching, granting of autonomy and teaching by pre-operative planning, relevant feedback and debriefing [49,50,51]. All these aspects, even though not RAS-specific, are relevant. The evidence regarding effective intraoperative RAS teaching is low. Therefore, further research examining the effect of teaching methods on intraoperative RAS skills is necessary. Rigorous objectifiable examination of teaching success measured by amount of trainee autonomy, intraoperative behaviour and patient outcome, will further clarify the skills and requirements for RAS surgical trainers and proctors. Additionally further examination of optimal modular training pathways specific to specialty and procedure should be undertaken.

## 5. Conclusions

This systematic review has identified the intraoperative RAS competencies required for surgeons in training. Important intraoperative skills are the use of the third arm, awareness of the operative field including off-screen activity, and management of the bedside team. The additional obstacles to teaching with the RAS console require teaching to be intentional and deliberate. Instructions are largely delivered by verbal interactions, which seem to be enhanced when accompanied by an illustrative gesture from the trainer. The novel teaching technique of “coming out of the console” may aid learning. Although training is possible without the dual consoles, it seems to be a beneficial investment for safe and successful intraoperative training. The quality of training could be improved by formally enhanced trainer teaching methods. The use of established training-the-trainer methods can be helpful for improving instructions, safely avoiding unnecessary takeover and promoting trainee autonomy.

## Figures and Tables

**Figure 1:**
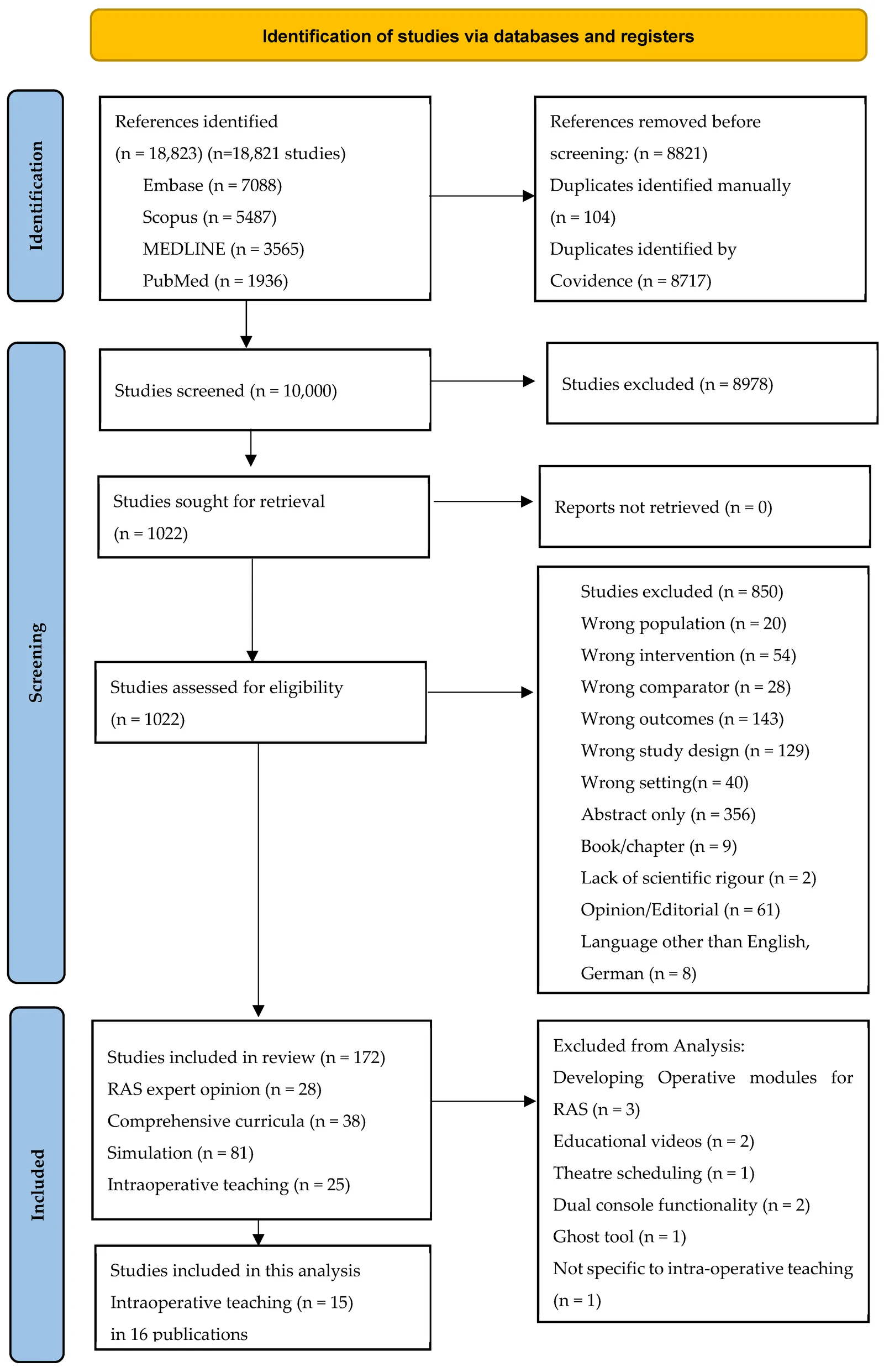
Flow chart of extraction process using PRISMA 2020 reporting methodology.

**Figure 2:**
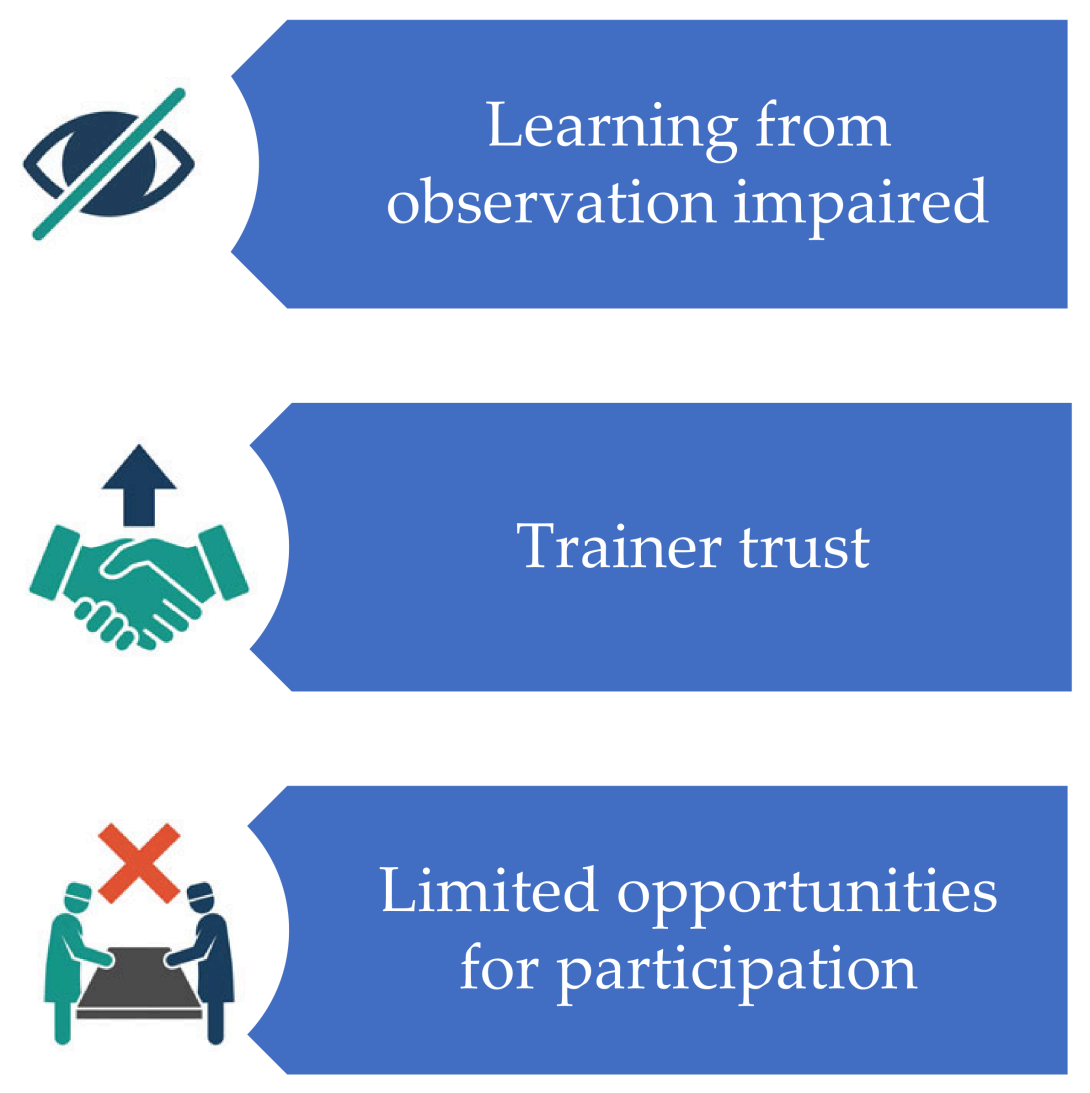
Barriers to RAS teaching.

**Figure 3:**
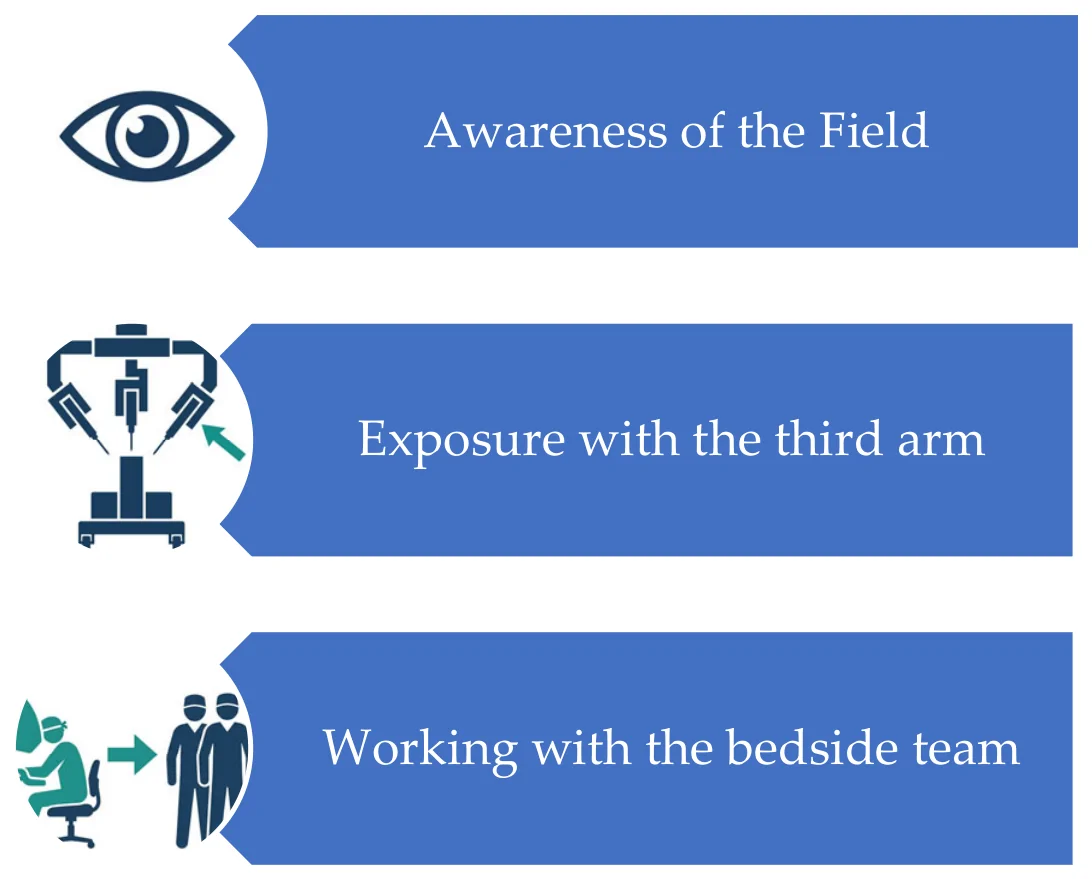
RAS skills learned in the intraoperative environment.

**Figure 4:**
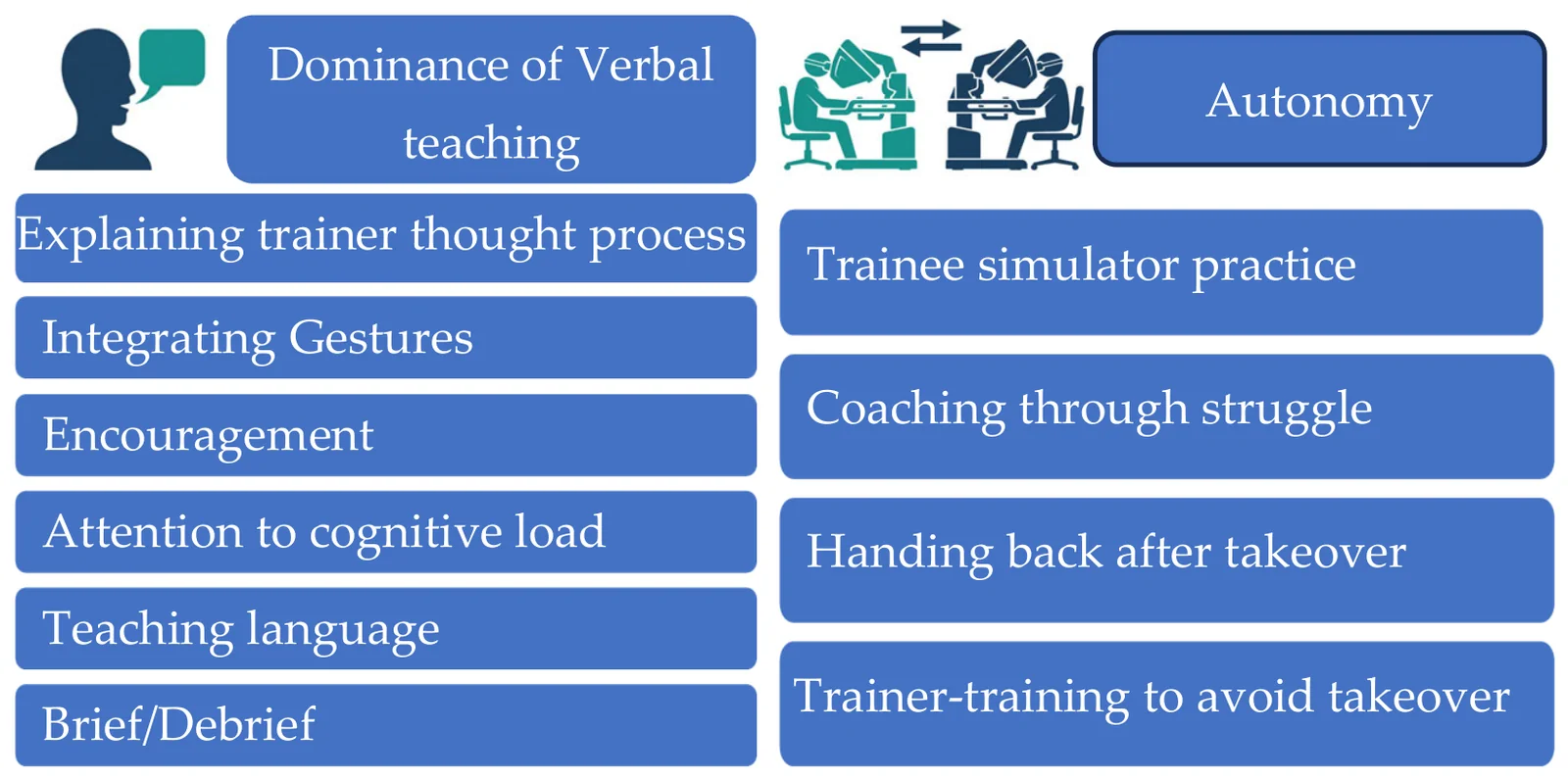
Teaching methods.

**Figure 5:**
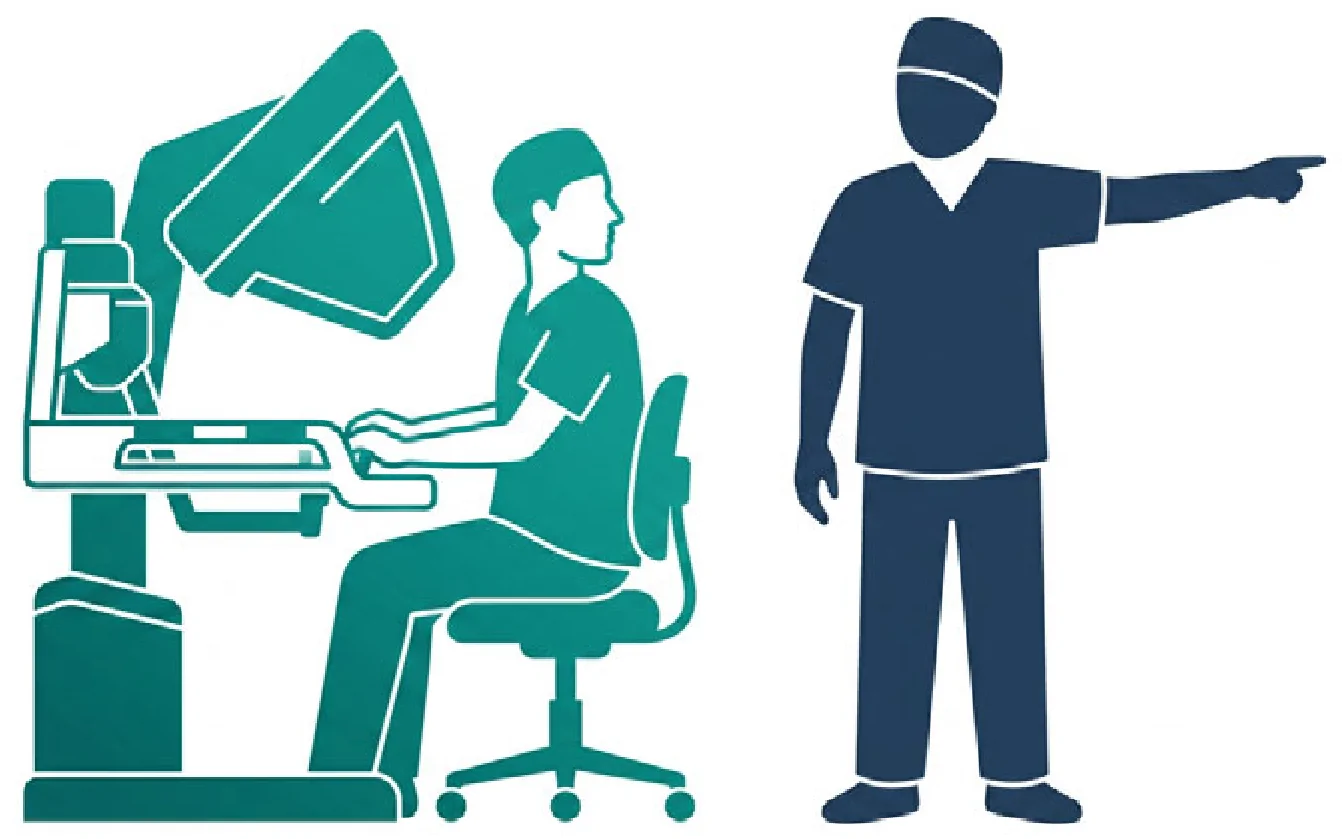
Disengaging from the console.

**Table 1:** PICO inclusion/exclusion criteria.

Eligibility Framework	Inclusion	Exclusion
Population	Surgeons, trainees	Medical students, other health professionals, established surgeons converting practice
Intervention	Intraoperative RAS teaching	Questionnaires, ultrashort interventions (single trial on simulator/one day course)
Comparator	Skill, engagement before/after intervention	Learning curve
Outcomes	Completion/engagement in curriculum, meeting predefined standard patient outcomes	Outcomes other than teaching/training of robotic surgery, e.g., simulator validation, feasibility of teaching, quality assessment mechanisms (crowd-based video review and robotic surgery assessment scores)
Setting	General Surgery, Urology, Gynaecology, Cardiothoracic surgery	Orthopaedic surgery, Ear Nose and Throat (ENT) Surgery, Neurosurgery
Publications	Peer-reviewed original publications	Abstracts, Books, Literature Reviews, Questionnaires, Editorials, single surgeon case series

## Data Availability

This is a review and no new data was collected. The reviewed publications are available as indicated in the references.

## Supplementary Materials

The following supporting information can be downloaded at: https://www.mdpi.com/article/10.3390/jcm15197529/s1, PRISMA_2020_checklist; File S1: Detailed platform specific search; Table S1: Examples from included studies.

## Abbreviations

The following abbreviations are used in this manuscript:

RAS	Robotic-assisted surgery
MMERSQI	Modified Medical Education Research Study Quality Instrument
JBI	Joanna Briggs critical appraisal tool
LapCo	Laparoscopic Colorectal Training the Trainer Course
ENT	Ear Nose and Throat
GEARS	Global Evaluative Assessment of Robotic Skills
GOALS	Global assessment of Laparoscopic skills
NASA TXL	National Aeronautics and Space Administration Task Load Index
