# Supplementary material for: Intraoperative Teaching in Robotic-Assisted Surgery—A Systematic Review
Source: J Clin Med. 2026 Sep 28;15(19):7529. doi: 10.3390/jcm15197529 (PMC13666273; doi:10.3390/jcm15197529)
Supplement: Supplementary file 1 [file jcm-15-07529-s001.zip › Tables S1 Examples from included studies.pdf]

Study	Findings	Examples	Credibility/Quality
Anand[43] et al. 2024 USA Differences in coaching in single-versus dual-console robotic cases: a mixed-methods study Multispecialty (61% Minimally invasive surgery/ small cases General surgery)	No difference found in resident autonomy score and faculty coaching score single vs. dual console<br>Majority of statements force sensitivity and robotic control<br>No evaluation of faculty<br>NASA TXL provided by console type<br>Gap in coaching self-reflection	Coaching<br>Single console: Domain Providing constructive feedback (n = 16, 30.2%) and Domain Guiding goal setting and creating actionable plan. (n = 18, 34.0%).<br>Dual Console: Domain Sharing responsibility. (n = 34, 37.0%) and Domain Guiding goal setting and creating actionable plan. (n = 33, 35.9%).<br>Pushback by trainees and faculty against console randomisation.<br>No examination of teaching which may have occurred pre-and postoperatively	Low
Beane[10] 2018 USA Shadow Learning: Building Robotic Surgical Skill When Approved Means Fail;<br>Urology	Barriers: no peripheral participation,<br>Visible mistakes,<br>Helicopter training by trainers, trainer independence from trainees<br>Successful trainees engaged in shadow learning: ‘‘premature specialization’’; ‘‘abstract rehearsal’’ (Simulation and video) ‘‘under-supervised struggle,’’ at the edge of their abilities	“There’s more going on [in open surgery than in robotic], it’s not apparent to everyone in the room, and it’s easier for the attending to also move in a different direction if you’re not doing something right, they can take . . . instead of 50–50, it can be a 75– 25 sort of thing, basically instead of letting you get into the appropriate plane of a dissection, they’re getting into the planes of the dissection and you’re basically just cutting. But you still feel like you’re part of the procedure and the attention is not pointed in a negative way. (Resident)”	Moderate ethnography without positionality of researcher
Brian et al.[47] 2014 USA Comparing observed and preferred	Suboptimal instructional practices contradicted preferred ways of learning were observed: Contextualisation	“I want someone to walk me through their thought process instead of ‘don’t do that’ and ‘don’t do this' because then I’ll stop doing what I’m doing, but I don’t know what the positive manoeuvre is” (P13)”	High

instruction in robotic surgery<br>General surgery	without explaining reason<br>Individualisation<br>without consideration of trainee level<br>Autonomy trainer cannot explain what is to be done and takes over<br>Multimodality -clarity is provided with addition of gesture	"The less familiar I am with the case, the more consistent ongoing feedback I want. And the more familiar I am with the case, I like it when they let me just struggle a little bit" (P15)"<br>"The attending is unable to explain verbally what he wants… (and) ends up taking control" (FN9)"<br>"Drawing sometimes is the most helpful because it just makes it so clear the plane that they want you to follow" (P11)"	
Christofari et al.[48]<br>2021<br>Switzerland<br>"Teaching and learning robotic surgery at the dual console: a video-based qualitative analysis"<br>Bariatric surgery	Skills described:<br>Managing the (bedside) team<br>Using the third arm<br>Perception of the operative field<br>Instructions:<br>Physical separation requires different instructions<br>More specific instructions necessary	S1: "What I find interesting is that we can also […] present the thread so that he can take it, or we can […] just hold it, and [S2] does it very often, she does not present it, she does not make life easier for the bedside assistant, and that's also a sign…. It's like when you're driving and you have to make things easy for people close by, for people further away. And [S2] is often very concentrated on what she's doing, but not yet on how to make the whole team work well… It's not a reproach, it's just a comment I'd say; these are things that will get better over time."	Moderate
Collins et al.[49]<br>2018 Europe<br>"Utilising the Delphi Process to Develop a Proficiency-based Progression Train-the-trainer Course	100% agreement of need of TTT course<br>Participants experts with clearly defined selection criteria<br>Surgical trainers should be knowledgeable, interested, and enthusiastic; enjoying training; being a good communicator; being supportive; having time to train; and knowing when to interject to prevent harm without trying to take over; know how to set up a standardised training program		Delphi, 80% agreement, 3 rounds, 82% (23/28) response rate first round, 89% (25/28) 2nd and 3rd rounds:

for Robotic Surgery Training”<br>Multispecialty<br>Participants from USA and eight European countries.	Delegates should be tested on the TTT course, proficiency of teaching topic before becoming “trainer”<br>Instruction on how to optimise guidance on defined technical skills, and training in providing feedback and debriefing following the assessment of technical skills<br>Trainer should always seek agreement with the trainee regarding the goals and objectives (trainee-directed learning).<br>The TTT course should provide opportunities to practice rating training skills in the operating room and laboratory/simulation setting<br>Understanding how to “shorten” the learning curve in surgery<br>Robotic surgery assessment includes technical skills, cognitive assessment, and nontechnical skills.		Cronbach alpha 0.90
Eardley et al.[50] 2020 UK<br>“European Society of Coloproctology Colorectal Robotic Surgery Training for the Trainers Course – the first pilot experience”<br>Colorectal<br>Train-the- Trainer	Three delegates received no previous train-the trainer teaching<br>Delegates increased their knowledge of each of the course learning objectives and identified learning points in order to change practice.<br>The issue of “no common language” was highlighted during the course and the question raised in the feedback questionnaire	3 most important things learned at the course: Hands off!‘ ‘Unconscious competence‘ ‘How to structure training: set, dialogue, closure, and the components of each part ‘ ‘Importance of feedback, how to provide it, and situation awareness ‘<br>Regarding common language:<br>‘Not something I thought about before coming to the course. I have seen many different training styles/languages used previously. Did wonder at some points in time whether people were talking about the same thing, for example, third arm, left hand etc. That’s something that we have to improve‘	MMERSQI 45
Gomez Ruiz et al.[51] 2019 Spain; “Expert consensus on a train-the-trainer	Trainers should all follow the same guidelines as defined in specialty specific curriculum.<br>Subject areas:<br>Conscious competency<br>Taking over in theatre		Delphi, 80% agreement 3 rounds, Response rate:<br>100%, 100%, 93%

curriculum for robotic colorectal surgery" Colorectal	Performance-enhancing instruction<br>Dual-task interference<br>Optimizing communications<br>Optimizing the training environment<br>Reflection on training<br>Dealing with difficult trainees<br>Optimizing feedback to the trainee<br>Maximizing dual-console teaching, theatre team training, nontechnical skills training, patient safety, user–machine interface training and telementoring.		Cronbach alpha >0.8
Green et al.[52] 2018 USA "Microanalysis of video from a robotic surgical procedure: implications for observational learning in the robotic environment" Colorectal	Observation of robot-assisted surgery is based on an incomplete visual framework<br>Change in operative focus occurs when there is an inability to obtain adequate tension<br>It may appear from video that single arm dissection occurs in RAS, this would vary from bimodal manipulation that occurs both in laparoscopic and open dissection.	Microanalysis revealed there was less than 1 min of [ ] third-party activity in a 15 minute video.[ ]Either one or two of the four robotic arms [are visible] on screen. However, when the camera zoomed out several minutes later, the [ ]third arm was functioning and intimately involved in the resulting on-screen actions [], even though its presence [ ]could not be visualized.<br>Changes in operative focus can be seen to have occurred immediately after increased activity of camera adjustment and tissue manipulation.<br>Bimodal manipulation is occurring in robotic surgery, but the action is at the console is not captured on screen. constant force appears motionless on the screen.	High
Green et al.[53] 2020 USA "Teaching in the robotic environment: Use of alternative approaches to guide	Observation:<br>Behaviours commonly used:<br>Verbal direction,<br>Explanation of thought process<br>Compliment	"I always struggle with the preciseness of language that is required to convey what you want and needs to be done [on the robot …] Like say for example you want [the resident] to flip the tissue. There is no good way to describe this … it's easy to describe like left, right, in, out, or	High

operative instruction"<br>General surgery	New teaching behaviour: disengaging the residents from the console to provide a teaching point<br><br>Themes from instructor focus group:<br>Importance of language.<br>Managing autonomy	instruments. But it's more difficult to describe subtle moves like rotate the mesentery out. I noticed a couple of times, you see, I was focusing on the monitor, but I wasn't sure what they were doing […] like how their hand positions were in the console. Like maybe their hands were far apart and they forgot to clutch and bring back. So I think the language is definitely hard […] You know if we don't have a common understanding of what I mean it can be hard too […] You definitely need to use a lot of language to really try to tell them what they need to do to help themselves."	
Green et al.[54] 2023<br>USA<br>"Enhancing robotic efficiency through the eyes of robotic surgeons: sub-analysis of the expertise in perception during robotic surgery (ExPeRtS) study"<br>General surgery	Areas for improved efficiency:<br>Case progression,<br>Robotic capabilities,<br>Instrumentation.	"This person seems to be a little bit…maybe ambivalent about some of the areas that they're working… tend to be moving around more than I would as far as working in one location. Almost like trying to decide if they feel like they're doing the right thing or not—Surgeon 14"<br>"I'm not sure where the third arm is. In fact, the third arm could be doing what the left hand now is doing so they can bring the left hand into help actually move this (operation) really along—Surgeon 4"<br>"So the technique… So the scissor tip open about one or two millimetres, you then use that feather technique. So you take the tip of the scissors, slide along the tissue, and it would feather open like that—Surgeon 8"	High

Green et al.[55] 2022 USA, Expertise in perception during robotic surgery (ExPeRtS): What we see and what we say General surgery	Visual comprehension: tissue structures, anatomic landmarks, adequate field of view.<br>Surgical technique: discussing dissection, tissue handling, instrumentation choice and exposure.<br>Risk Avoidance: Overarching theme for visual comprehension and surgical technique	"Wow, that, I don't like that at all, that's dangerous what they're doing. Totally buried that instrument, they can't see because the camera is totally in line, I don't know where these ports are placed, but it's not where I would've put them. You don't want them all to be in line like this and they have no ide … they're gonna dig right into the liver. Ay-yi-yi, that, I would not permit that. (Surgeon 4)"<br>"If they're going to take the duct like this, then it needs to be better dissected out. We need to know clearly that it's a ductal structure, that's clearly going directly into the gallbladder, and that there is a clear view, clear view behind it that shows that it's not, you know, common bowel tented up or something (Surgeon 11)"	High
Harji et al.[30] 2025 UK+ Ireland "Scaling robotic surgery: the role, responsibilities and challenges of robotic proctorship in colorectal surgery" Colorectal surgery	Proctor selection is currently undertaken and driven by industry partners and representatives, based on arbitrary criteria.<br>The aim of robotic proctoring is not to provide clinical or operative training.<br>Responsibility of the proctor, from an industry perspective, is to ensure safe use of the robotic technology<br>Proctors assume they have responsibilities: clinical, patient safety and training.<br>Position as an industry appointed proctor often allows them to have a wider sphere of influence within the surgical community in the delivery and direction of robotic training due to their overall experience.<br>The difficulty with 'sign off' and accreditation with the proctoring process is the small number of supervised cases i.e. 1–5 and the types of cases supervised.[…] Difficulties in the sign off process arise when the basic robotic platform related competencies have been achieved, however, the overall robotic operative standard is considered to be suboptimal.'		Moderate

	Proctors face a unique set of challenges, which are environmental, interpersonal, clinical, ethical, and medicolegal.<br>Unfamiliar clinical setting, with an unknown theatre set up and team, and limited knowledge and understanding of the training surgeons' operative capabilities and prior experience<br>Poor case selection by the training surgeon often compounds these challenges. Interpersonal challenges are related to differences in opinion, personality clashes between proctors and training surgeons, and the delivery of feedback<br>The robotic proctors are selected and remunerated by industry partners to deliver platform related training. Given the financial relationship between the proctor and the robotic platform provider, the priorities and objectives of both parties are expected to align.		
Leon et al.[56] 2022 USA "Impact of robotic single and dual console systems in the training of minimally invasive gynecology surgery (MIGS) fellows" Gynaecology	There were no significant differences between the two groups regarding docking time (P=0.15), case time (P=0.79), or complications (P=0.30).<br>Evidence of a significantly longer fellow console time (P < 0.001, Fig. 1), a higher console time/docking time ratio (P < 0.001, Fig. 2), a greater number of steps performed (P = 0.009), and a greater number of control switches between the fellow and attending (P < 0.001) for dual console patients compared to single console patients.		MMERSQI 53.5/85
Satchidanand et al.[57] 2020 USA "Put the what, where? Cut here?!" challenges to coordinating attention in robot-assisted surgery: a	Referencing strategies for communication were:<br>Verbal:<br>Anatomy/directional<br>Context dependent<br>Gesture:<br>Point/show<br>Functional movement<br>Camera focus movement	Analysis of referencing strategies, revealed that Integrated communication was the most frequently used (45%, n=21) and was the most effective strategy with 90% (n=19) of references successfully completed (binomial test, p<0.01);<br><br>"In 'Cut Where?' the trainee surgeon moved his scissors slowly into place before	High

microanalytic pilot study" Urology	Integrated communication: using verbal communication with gestures<br>The use of microanalysis revealed causal factors of miscommunication, including temporal misalignment	making his erroneous cut, leaving time both to request and be given instruction. Mentor surgeons and trainees can be made aware of opportunities like these and of the value of pausing in order to maintain temporal coordination."	
Wang et al.[58] 2024 USA "Behind the pattern: General surgery resident autonomy in robotic surgery"<br>General surgery	Residents may get less active time in RAS then they would be getting with other modalities. Resident ICT significantly increased with seniority (PGY level): on average, junior residents were allowed 26.8 % ICT per case vs. 42.4 % for senior residents ($p < 0.001$). ICT also varied significantly based on procedure type; attending surgeon work experience. Surgeon assessment of resident training year/experience influenced decisions to grant ICT; Surgeons' perceived operative time pressure inversely affected resident ICT.	"I also want the resident to struggle [in the OR] so the resident can learn, but with limited time and the crunch that I am going over my block time, I have to move the case along so sometimes I end up taking the case over" [Excerpt 5.1]<br>"If I'll have a new PGY2 [resident] who has done just a couple lap choles, and I don't know what this resident is gonna do, and I'm not sure if the resident would do something terrible, whether I can fix it. [Excerpt 2.1]"<br>"Operations can be broken down into steps, and I'm constantly checking the clock to say this step, we should have progressed by this stage if we aren't there yet, it's probably time for me to step in and give a hint or do that part of the case." [Excerpt 4.1]	High
Wong et al.[59] 2023 USA<br>"Development of a Classification System	Trigger: Error of omission, error of commission, warning, good trainee action, trainee question	Technical feedback provided with a visual aid component (e.g. the trainer saying, "Take a bigger bite so you get some mucosa" while simultaneously pointing	High

for Live Surgical Feedback” Urology	Feedback: Anatomic, Procedural, technical, visual ais, technical, praise, criticism<br>Trainee Response: verbal acknowledgement, behavioural change, asks for clarification<br>Trainer Response: approval, disapproval, repeats feedback (identical) repeats feedback (similar), takes over (non-safety) takes over (for safety)<br>Combinations of trainer feedback had associations with different responses.	with the telestrator at the mucosal edge) was found to have a significantly increased rate of trainee behavioural change or verbal acknowledgment responses compared with no technical feedback (RR, 1.11 [95% CI, 1.03-1.20]; P = .02). Providing technical feedback without visual aid did not have a statistically significant association with trainee behavioural change or verbal acknowledgment responses (RR, 1.01 [95% CI, 0.93-1.10]; P = .73).
